# Pharmaceutical Strategies to Improve Druggability of Potential Drug Candidates in Nonalcoholic Fatty Liver Disease Therapy

**DOI:** 10.3390/pharmaceutics15071963

**Published:** 2023-07-16

**Authors:** Reeju Amatya, Donghee Lee, Kyoung Ah Min, Meong Cheol Shin

**Affiliations:** 1 College of Pharmacy and Research Institute of Pharmaceutical Sciences, Gyeongsang National University, 501 Jinju Daero, Jinju 52828, Republic of Korea; reejuamatya94@gmail.com (R.A.); dhdl3543@daum.net (D.L.); 2 College of Pharmacy and Inje Institute of Pharmaceutical Sciences and Research, Inje University, 197 Injero, Gimhae 50834, Republic of Korea

**Keywords:** nonalcoholic fatty liver disease, nonalcoholic steatohepatitis, fibrosis, cirrhosis, strategies

## Abstract

Nonalcoholic fatty liver disease (NAFLD) has become globally prevalent and is the leading cause of chronic liver disease. Although NAFLD is reversible without medical intervention in the early stage, the condition could be sequentially worsened to nonalcoholic steatohepatitis (NASH) and, eventually, cirrhosis and hepatic cancer. The progression of NAFLD is related to various factors such as genetics, pre-disposed metabolic disorders, and immunologic factors. Thankfully, to date, there have been accumulating research efforts and, as a result, different classes of potent drug candidates have been discovered. In addition, there have also been various attempts to explore pharmaceutical strategies to improve the druggability of drug candidates. In this review, we provided a brief overview of the drug candidates that have undergone clinical trials. In the latter part, strategies for developing better drugs are discussed.

## 1. Introduction

NAFLD is a condition characterized by hepatic fat accumulation without chronic alcohol consumption or the use of steatogenic drugs. NAFLD is a relatively broad term comprising nonalcoholic fatty liver (NAFL) to stages when chronic fat accumulation results in hepatic inflammation and injury, engendering NASH and cirrhosis [1]. Currently, NAFLD is the most common liver disease worldwide, with a global prevalence of 25%. Various reports have indicated that people with progressing age and conditions such as type 2 diabetes (T2DM), hypertension, obesity, and dyslipidemia are high-risk groups for developing NAFLD [2,3,4]. For example, the prevalence of NAFLD increases to over 90% in obese people and 60% in T2DM patients [5].

NAFLD is diagnosed when hepatic steatosis is detected upon abdominal imaging, which could be accompanied with or without elevation in the levels of alanine and aspartate aminotransferases (ALT and AST) in blood. NASH is diagnosed upon liver biopsy, if hepatocellular injury (ballooning) and inflammation are detected in the presence or absence of fibrosis [6,7]. Hepatic steatosis can be detected with the help of noninvasive imaging tests such as magnetic resonance imaging (MRI) and computed tomography (CT). MRI is more sensitive and expensive than CT in detecting steatosis, and can detect steatosis when it is as small as 5% of liver mass [7]. The NAFLD activity score (NAS) can be used to score the histological features upon biopsy by determining the presence of an anomaly. The NAS ≥ 5 has been correlated with the presence of NASH. However, there is a lack of reliable biomarkers for NALFD/NASH in the early stages. The stages of NASH can be determined via the fibrosis-4 index (FIB-4) or NAFLD fibrosis score (NFS). The probability of developing fibrosis can be determined using the NFS. If the NFS is <−1.5, it is called “low probability”; between −1.5–0.65, “intermediate probability”; and >0.65, “high probability” for fibrosis [8]. Upon histological examination, liver fibrosis can be classified as F0 (absence of fibrosis), F1 (perisinusoidal fibrosis), F2 (portal/periportal fibrosis), F3 (bridging fibrosis), and F4 (cirrhosis) [9]. According to studies, 41.9% of NAFL patients progressed to NASH, and 30% of NASH patients progressed to fibrosis. Approximately 22% of NASH patients with F3 stage fibrosis progressed to cirrhosis [10]. Recently, ‘multiple-hit hypothesis’ has been put forward to explain the pathogenesis and progression of NAFLD. The pathogenesis of NAFLD starts with excessive accumulation of free fatty acids (FFAs), cholesterol, and triglycerides (TGs) in the hepatocytes, overwhelming the liver’s function in maintaining lipid homeostasis [11]. The liver is originally not a fat storage organ, and there is, generally, some FFA trafficking between the liver and the body [12]. Rather than being stored in the liver, the FFAs in the liver would go through β-oxidation (in mitochondria), or be secreted into the plasma after being converted to TG and packaged to form VLDL. However, if an imbalance between lipid acquisition and deposal occurs, fat will accumulate in the liver, which is the hallmark of NAFLD. In NAFLD, lipid droplets are formed in hepatocytes, a characteristic feature of NAFLD. Dysfunction of adipocytes with uncontrolled hepatic de novo lipogenesis (DNL) and decreased lipolysis lead to hepatic steatosis [13]. The unrestrained hepatic accumulation of TGs and FFAs causes the generation of oxidative stress, mitochondrial dysfunction, endoplasmic reticulum (ER) stress, and hepatocellular damage [11]. The major non-parenchymal cells in the liver, sinusoidal endothelial cells (LSECs), and hepatic stellate cells (HSCs), usually serve for regeneration and recovery of the liver from inflammation and injury. However, capillarization and de-differentiation of LSECs are critical events in the progression of liver fibrosis. The dysfunction of LSECs also promotes inflammatory pathways and steatosis [14]. Compromised hepatocyte integrity and activation of inflammatory pathways—such as c-Jun N-terminal kinase-1 (JNK1) and the nuclear factor kappa B (NFκB) signaling pathways—with suppression of peroxisome proliferator-activated receptor-α (PPAR-α) could induce trans-differentiation of quiescent HSCs into myofibroblast-like activated HSCs (aHSCs) [15,16]. Upon activation, the HSCs lose the storage of vitamin A and become proliferative. They also show increased expression of smooth muscle α-actin (SMA) and transforming growth factor-β1 (TGF-β1), which are primarily responsible for the deposition of type I collagen in the extracellular matrix (ECM) [17]. Hepatocellular ballooning and lobular inflammation with the absence or presence of a certain degree of fibrosis are the primary features of NASH. Long-term injury and inflammation can induce hepatic fibrosis and, in extreme cases, further advance to cirrhosis, which is the condition of excessive scarring with impaired liver function.

Other factors contribute to the progression of NAFLD. In the gut, inflammasomes are essential for microbiota homeostasis, the expression of microbial peptides, and the regulation of immune response. Hence, the lack of inflammasomes in the gut may result in high expression of pro-inflammatory cytokines (e.g., toll-like receptor-4 (TLR-4) and TLR-9) and exacerbate NASH [18]. There are also genetic risk factors that could affect the progression of NASH. The polymorphism in the *PNPLA3* gene regulating hepatocellular lipolysis, splice variant (rs72613567:TA) in the HSD17B13 gene, and impairment of TM6SF2 gene are related to increased NAFLD development [7]. Insulin resistance is also a critical factor exacerbating the dysfunction of lipid metabolism and homeostasis. Another pathogenic factor could be the altered gut microbiome and gut-liver axis attributable to the modern diet that is high in saturated fat, carbohydrates, and a sedentary lifestyle. However, the pathological drivers may vary among the patients, as multiple molecular pathways are involved in the disease’s progression. As a result of the involvement of metabolic abnormalities and a broad spectrum of complex phenotypes and epigenetic factors, NAFLD is also called “metabolic-dysfunction associated fatty liver disease (MAFLD)” [19].

The global prevalence of NAFLD and its rapid increase urgently demand the discovery of effective drugs, as well as elucidation of the pathological mechanism. The early chapter of this review will introduce a relevant class of drug candidates that have undergone clinical investigation, while the latter part will cover the trends of current pharmaceutical strategies explored to make “better drugs”.

## 2. Therapeutic Drug Candidates for the Treatment of NAFLD

Unfortunately, there are very limited treatment options for NAFLD therapy [20]. The recommended first-line treatment includes dietary and lifestyle modification. Antioxidants such as vitamin E and glutathione (GSH) are also listed as the first-line treatment for patients without T2DM [21]. In addition, surgical treatments are available. Bariatric surgery could improve the metabolic condition and survival rate, and liver transplantation remains the last treatment option for NASH management [22,23]. However, there is currently no approved pharmacotherapy for NAFLD [24]. This lack of available drugs could be explained by several reasons. First, NAFLD is a complex disease that encompasses a broad spectrum of conditions that include simple steatosis, NASH, and fibrosis. During the progression of the disease, various metabolic and immunogenic factors are involved, which may require multifunctional effects by the drugs or combination therapies. Second, the underlying mechanism of the pathogenesis of NAFLD is in progress, but the related key factors and appropriate drug targets are not yet fully identified. Lastly, there are great challenges in clinical trials, because of the slow, long-term progression of the disease. The significant therapeutic effects of the drug candidates should last for a long period (probably more than a year). Many drug candidates have failed in clinical trials, mainly because of a lack of sufficient efficacy or potential toxicity concerns.

**Figure 1:**
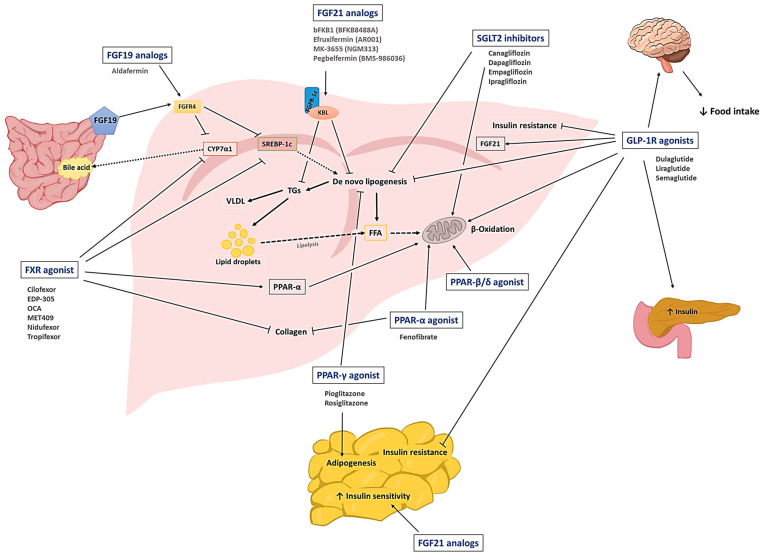
Schematic representation of crosstalk between the organs and major regulatory pathways in NAFLD. A group of NAFLD drug candidates (e.g., SGLT2 inhibitors, GLP-1R agonists, PPAR agonists, FGF19 and FGF21 analogs, and FXR agonists) have been developed and have undergone (or are still ongoing) clinical trials. The action mechanisms of these compounds involve various and multiple pathways related to: (1) regulation of glucose and lipid homeostasis in the network of adipose tissues, the brain, pancreas, and liver; (2) modulating de novo lipogenesis, lipolysis, and β-oxidation of free fatty acids; (3) reducing food intake (indicated by downward arrow) and body weight; (4) improving insulin resistance (increased insulin sensitivity indicated by upward arrow) in liver and adipose tissues; and (5) regulating bile acid production (CYP7α1, cholesterol 7-α-hydroxylase; FFA, free fatty acid; FGF, fibroblast growth factors; FGFR, fibroblast growth factor receptor; FXR, farnesoid X receptor; GLP-1R, glucagon-like peptide-1 receptor; PPAR, peroxisome proliferator-activator receptor; KBL, β-klotho; SGLT2, sodium-glucose co-transporter 2; SREBP-1, sterol regulatory element-binding protein-1; TGs, triglycerides; VLDL, very low density lipoprotein).

Despite the challenges, there have been accumulating studies to discover novel drug candidates for treating NAFLD. The main mechanisms of these drugs generally include: (1) modulating glucose and lipid homeostasis, (2) reducing hepatic injury and inflammation, and (3) ameliorating fibrosis [20]. In this chapter, we will introduce a group of representative drug candidates that have undergone extensive pre-clinical and clinical investigations. The class of drug candidates includes sodium-glucose co-transport protein-2 (SGLT2) inhibitors, glucagon-like peptide-1 receptor (GLP-1R) agonists, peroxisome proliferator-activated receptors (PPAR) agonists, fibroblast growth factors (FGFs), and farnesoid X receptor (FXR) agonists. A schematic illustration of the drugs and their primary mode of action is shown in Figure 1.

### 2.1. SGLT2 Inhibitors

The SGLT2 inhibitors are clinically available anti-hyperglycemic agents that limit renal glucose reabsorption. They have been approved for the treatment of heart failure and chronic renal diseases [25]. Currently, there are a total of eight SGLT2 inhibitors approved worldwide: dapagliflozin, canagliflozin, empagliflozin, and ertugliflozin (in the U.S.), luseogliflozin and topogliflozin (in Japan), ipragliflozin (in Japan and Russia), and remogliflozin etabonate (in India) [25]. Apart from being anti-diabetic drugs, SGLT2 inhibitors are potentially effective drug candidates for the treatment of NAFLD. They can reduce the hepatic lipid content by inhibiting de novo lipogenesis (DNL) [26] and promoting the β-oxidation of FFAs [27]. Moreover, they can reduce bodyweight, and provide anti-oxidative and anti-inflammatory effects [25]. For example, in *ob/ob* and HFD-fed obese mice, ipragliflozin significantly reduced DNL and hepatic steatosis, and improved insulin resistance [28]. The effects of ipraglifozin were also revealed in clinical studies. In a clinical trial with T2DM patients with NAFLD (title: Ipragliflozin for type 2 DM with NAFLD, ID: UMIN00001572, phase: N/A), 72-week treatment of ipraglifozin could reverse NAFLD in 67% of the patients, with no development of NASH. Notably, this study’s results implied that ipragliflozin may also help prevent the development of NASH [29]. In another randomized controlled trial (Effects of Ipragliflozin on Excessive Fat in Type 2 Diabetes Patients With Non-alcoholic Fatty Liver Disease Treated With Metformin and Pioglitazone, NCT02875821, phase 4), a 24-week treatment of ipragliflozin with pioglitazone and metformin in diabetic NAFLD patients provided a significant reduction of hepatic and whole-body visceral fat contents [30]. According to a meta-analysis of 11 clinical trials, dapagliflozin provided a significantly higher reduction of ALT, AST, γ-glutamyl transferase (GGT), TG, bodyweight, body mass index (BMI), HbA1c, and fasting plasma glucose levels than the control groups [31]. In a different report (A Study to Investigate Effects of Omega-3 Carboxylic Acids and Dapagliflozin on Liver Fat Content in Diabetic Patients (EFFECTII), NCT02279407, phase 2), combination therapy with dapagliflozin and omega-3 carboxylic acid more significantly reduced liver fat content than the monotherapy groups [32]. Other SGLT2 inhibitors such as luseogliflozin (Effective and safe examination of Luseogliflozin for patients with fatty liver merger type II diabetes mellitus, UMIN000021087, phase N/A) [33], empagliflozin (Effect of Empagliflozin on Liver Fat Content in Patients With Type 2 Diabetes (E-LIFT), NCT02686476, phase N/A) [34], and canagliflozin (Efficacy of canagliflozin against nonalcoholic fatty liver disease, phase N/A) [35] were also effective in reducing hepatic fat content, transaminase activity, and HbA1c level. The active clinical studies of SGLT2 for NAFLD are summarized in Table 1.

### 2.2. GLP-1R Agonists

GLP-1 is an incretin hormone released from the enteroendocrine L-cells. It exhibits a broad spectrum of activities, including regulation of insulin signaling, glucose metabolism, satiety signals, and bodyweight [36]. Regarding NAFLD, GLP-1R agonists are reportedly capable of alleviating hepatic lipotoxicity and inflammation through various cell signaling pathways (e.g., insulin receptor substrate-2 (IRS2)/phosphatidylinositol-3 kinase (PI3K)/Akt [37] and mTORC1 [38] signaling pathways). They can also modulate Ca^2+^ signaling and reverse hepatic insulin resistance [39].

There have been extensive pre-clinical and clinical study reports of long-activing GLP-1R agonists’ (e.g., dulaglutide, liraglutide, and semaglutide) potential use in the treatment of NAFLD [40]. For example, in a rapid-onset NASH mice model, liraglutide treatment significantly reduced hepatic steatosis, inflammation, and TG levels with improved glucose metabolism and insulin resistance [41]. Also, in methionine and choline-deficient (MCD) diet-fed mice, liraglutide could prevent hepatic inflammation and the development of fibrosis [42]. Similar to liraglutide, semaglutide reduced hepatic DNL and inflammation, improved insulin sensitivity, and promoted β-oxidation of FFAs in obese mice [43]. In clinical trials, liraglutide was well tolerated in NASH patients and reduced hepatic DNL, and improved insulin sensitivity in hepatic and adipose tissues (Liraglutide Efficacy and Action in Non-Alcoholic Steatohepatitis (LEAN), NCT01237119, phase 2) [44,45]. According to a meta-analysis, liraglutide significantly improved the aminotransferase, lipoprotein, and adipose tissue levels among NAFLD patients and reduced BMI and TG levels [46]. Semaglutide also reduced bodyweight, ALT, and high sensitivity C-reactive protein (hsCRP) levels in obese diabetic patients (Investigation of Safety and Efficacy of Once-daily Semaglutide in Obese Subjects Without Diabetes Mellitus, NCT02453711, phase 2) [47]. A systemic review of clinical trials revealed that both liraglutide and semaglutide significantly reduced hepatic fat accumulation and attenuated the worsening of hepatic fibrosis [48]. However, a recent trial result showed that semaglutide (once weekly) is more effective than liraglutide (once daily) in reducing bodyweight in obese but not diabetic patients (Research Study to Investigate How Well Semaglutide Works Compared to Liraglutide in People Living With Overweight or Obesity (STEP 8), NCT04074161, phase 3) [49]. Ongoing clinical trials of GLP-1R agonists for NAFLD are summarized in Table 2.

### 2.3. PPAR Agonists

PPARs are a group of nuclear receptors that serve as transcription factors in lipid metabolism, the regulation of inflammation, and glucose homeostasis [50]. Three isoforms of PPARs (PPAR-α, PPAR-β/δ, and PPAR-γ) have been reported. Specifically, the PPAR-α agonists (typically known as “fibrates”) are clinically available anti-hyperlipidemic agents, while the PPAR-γ agonist, thiazolidinedione, is an anti-hyperglycemic agent for T2DM management. Despite differences in tissue distribution, ligands, and physiological characteristics, all three isoforms of PPARs are commonly involved in lipid metabolism [51]. The PPAR-α is the most abundant isoform in metabolically dynamic tissues such as the heart, brown adipose tissues, and the intestinal mucosa (but is mainly present in the liver and in skeletal muscle), where it participates in lipid oxidation and metabolism [51]. The PPAR-γ is predominantly expressed in brown and white adipose tissues and is involved in lipid biosynthesis, adipogenesis, insulin sensitivity, and energy homeostasis [51]. The isoform PPAR-β/δ is ubiquitously present throughout many tissues, but is primarily expressed in liver and abdominal adipose tissues, where it modulates β-oxidation of FFAs, blood lipid levels, and glucose homeostasis [52].

PPAR agonists have been examined for their effects on NAFLD [50]. Specifically, pioglitazone has been of great interest, and a total of 30 clinical studies have been carried out (or are still ongoing). In a clinical trial, pioglitazone improved insulin sensitivity, NAS score, fibrosis score, and TG levels in patients with T2DM and biopsy-proven NASH (University of Texas H.S.C. San Antonio Pioglitazone in Non-Alcoholic Steatohepatitis Trial (UTHSCSA NASH Trial), NCT00994682, phase 4). Similarly, according to a meta-analysis of clinical trials, pioglitazone was found able to significantly improve steatosis, inflammation, ballooning grades, fasting glucose, and insulin levels. However, pioglitazone was ineffective for fibrosis [53]. There have also been clinical trials with rosiglitazone. In a 1-year randomized trial, rosiglitazone improved steatosis and ALT levels in NASH patients (A One-Year, Randomized, Double-Blind, Placebo-Controlled Trial of Rosiglitazone in Non-Alcoholic Steatohepatitis (FLIRT), NCT00492700, phase 2) [54]. However, additional 2-year treatment with the rosiglitazone could not significantly improve the conditions of steatosis or fibrosis. In the case of rosiglitazone, there have been placed restrictions by the FDA, due to significant cardiovascular risk issues [55]. Fenofibrate was also clinically investigated but did not meet the criteria (A Double-blind Randomized Placebo-controlled Study Comparing Epanova and Fenofibrate on Liver Fat in Overweight Subjects, NCT02354976, phase 2). Among the PPAR agonists, there are currently four active clinical trials of pioglitazone for NAFLD. They are summarized in Table 3.

### 2.4. Fibroblast Growth Factor (FGF) Analogs

The FGFs are a family of polypeptide ligands for the transcriptional nuclear FGF receptors (FGFRs). All of them share structural similarities and have multifunctional roles in development. Among the 22 known FGFs, FGF19 and FGF21 are engaged in maintaining lipid and glucose homeostasis [56,57]. The FGF21 is predominantly present in the liver, but is also expressed in adipose tissues [58]. In the adipose tissues, the FGF21 induces insulin-independent glucose uptake [58]. Hence, FGF21 could reduce blood glucose and TG levels in various diabetic mice models. According to Wang et al., the FGF21 treatment for monosodium glutamate (MSG)-induced obesity rats could not only reduce the bodyweight, fat mass, and blood glucose level, but also significantly improve liver function and inflammation [59]. The following studies have proven that FGF21 is potentially effective in improving NAFLD through various mechanisms that involve reducing hepatic DNL, inflammation, the production of cholesterol and bile acids, and promoting glucose metabolism and glucose uptake by adipocyte tissues [60]. From FGF21-transgenic mice, significant side effects (e.g., stunted growth, female infertility, bone loss, and increased serum glucocorticoid levels) were also recognized, suggesting the necessity of effective yet safer FGFR agonists [61]. In this regard, several bispecific antibodies have been developed against the FGFR-1/β-klotho complex as potential therapeutics for the treatment of obesity-related metabolic diseases. The bFKB1 (also named “BKFB8488A”), a humanized FGFR-1c/β-klotho complex bispecific antibody developed by Kolumam et al., revealed the specific activation of only the FGFR-1c, different from the FGF21 (that activates FGFR-1c, 2c, and 3c) and had the least interference with the binding of endogenous FGFs (FGF19 and FGF21) to the FGFRs. Notably, sustained metabolic effects (improvement in bodyweight, hepatic steatosis, hyperlipidemia, and hyperglycemia) were obtained by a single administration of bFKB1, which was equivalent to repeated or continuous infusion of the FGF21 in the tested mice (HFD-fed DIO and *db/db*) and cynomolgus monkey models. More importantly, side effects observed from FGF21 and FGF19 (such as increased corticosteroid levels and hepatic cell proliferation) were not found in the bFKB1-treated DIO mice [61]. Recently, a 12-week phase 1 clinical trial (A Multiple Ascending Dose Study to Evaluate Safety and Tolerability of BFKB8488A in Participants with Type 2 Diabetes Mellitus and Participants with Non-Alcoholic Fatty Liver Disease, NCT03060538, phase 1) for the bFKB1 has been completed. The bFKB1 was proven safe and could significantly reduce the TG, ALT, AST, and liver fat content in NAFLD patients [62]. The MK-3655 (NGM313) is another humanized monoclonal antibody against the FGFR-1c/β-klotho complex. Compared with pioglitazone, the MK-3655 exhibited a significantly higher reduction in TG, LDL-c, and HAb1c levels and liver fat contents (Study of NGM313 in Obese Participants, NCT03298464, phase 1) [63]. It is currently under investigation in a phase 2 clinical trial (A Study of MK-3655 in Individuals with Pre-cirrhotic Nonalcoholic Steatohepatitis (NASH) (MK-3655-001), NCT04583423, phase 2).

FGF19 is mainly involved in the regulation of bile acid synthesis. The luminal bile salt in the ileum induces the FGF19 expression. The FGF19 signals through its binding to the β-Klotho-FGFR4 and inhibits bile acid synthesis by suppressing the expression of cholesterol 7α-hydroxylase (CYP7A1) [64]. In the report by Wunsch et al., the levels of FGF19 in the serum and liver were found to correlate with the severity of cholestatic liver diseases such as primary biliary cirrhosis [65]. FGF19 dysregulation has also been reported in NAFLD, but interestingly, a reduced fasting FGF19 was correlated with the development of steatosis in obese adolescents [66]. Hence, FGF19 has been considered a potential drug candidate for treating NAFLD. However, the pro-tumorigenic activity of FGF19 remained a major concern [65]. To address this issue, the development of non-tumorigenic FGF19 analogs has been actively pursued. For example, a genetically engineered FGF19-M52 protein constructed by Gadaleta et al. retained the intrinsic properties of FGF19 without being tumorigenic. The FGF19-M52 treatment of Abcb4^−/−^ and fxr^−/−^ mice could protect mice from hepatic collagen deposition, liver damage, and spontaneous fibrosis [67]. Aldafermin (NGM282), another non-tumorigenic FGF19 analog, induced a significant reduction in hepatic fat content (≥30%) and improvement of NAS score (with two or more points) in patients after a 12-week treatment without worsening of NASH and fibrosis (Study of NGM282 in Patients With Nonalcoholic Steatohepatitis (NASH), NCT02443116, phase 2) [68]. Two 24-week clinical studies with aldafermin (Evaluation of Efficacy, Safety and Tolerability of Aldafermin in a Phase 2b, Randomized, Double-blind, Placebo-controlled, Multi-center Study In Subjects With Nonalcoholic Steatohepatitis and Stage 2/3 Fibrosis (ALPINE 2/3), NCT03912532, phase 2 and Study of NGM282 in Patients With Nonalcoholic Steatohepatitis (NASH), NCT02443116, phase 2) showed a significant reduction of hepatic fat, ALT, AST, and Pro-C3 levels with improvement in hepatic fibrosis. However, an increase in cholesterol level was also observed as a side effect. In this regard, co-administration of a cholesterol-modulating agent (such as statins) was considered beneficial [69]. The FGF analogs in active clinical trials for NAFLD are summarized in Table 4.

### 2.5. FXR Agonists

The nuclear receptor FXR is a transcription factor highly expressed in the liver and intestine. It serves as a bile acid sensor and is involved in bile acid metabolism and the regulation of cholesterol and TGs [70]. The deficiency of FXR in mice was related to NASH-like pathology with increased hepatic steatosis, ballooning, and inflammation [71]. The activation of FXR can (1) indirectly suppress the transcription of CYP7A1 via regulation of the FGF19 gene [70]; (2) induce the inhibition of the SREBP1-c regulated fatty acid synthesis by activation of an atypical nuclear receptor heterodimer partner, SHP (small heterodimer partner; alternative name: NR0B2) [72]; and (3) promote the expression of PPARα, leading to the promotion of FFA oxidation [73]. Thus, the FXR agonists are likely to exhibit a dual action to improve NAFLD by inhibiting lipogenesis (via the crosstalk with SREBP-1c) and promoting lipolysis (via activation of the FXR-PPARα pathway) [74]. Owing to these beneficiary actions through the FXR, many FXR agonists have been investigated for their potential use in NAFLD treatment.

A recent trend in the development of FXR agonists is the discovery of potent molecules that cause less adverse effects, as the treatment of early FXR agonists (e.g., bile acids and primary bile acid analogs) were often accompanied by pruritus, the elevation of LDL cholesterol levels, and liver toxicity. It was found that the pruritus might be caused by the activation of GPBAR1 (alternative names: TGR5, M-BAR, BG-37, hGPCR19, and AXOR 109) [75,76]. Hence, many research efforts have been made to develop more FXR-selective bile acid-based agonists. A representative example is the obeticholic acid (OCA, alternatively, 6α-ethyl-chenodeoxycholic acid or INT-747), a semi-synthetic chenodeoxycholic acid (CDCA) variant. It is a potent and selective FXR agonist (EC_50_: 99 nM) [77] with 100 times higher affinity than the endogenous CDCA [78]. In pre-clinical studies with NAFLD mice models (melanocortin 4-receptor deficient (MC4R-KO) NASH mice and western diet-fed, CCl_4_-treated mice), the OCA improved insulin resistance, liver steatosis, and fibrosis [79,80]. OCA’s therapeutic efficacy for NAFLD has been further investigated in clinical trials. In a 72-week study (The Farnesoid X Receptor (FXR) Ligand Obeticholic Acid in NASH Treatment Trial (FLINT), NCT01265498, phase 2), the OCA treatment for NASH patients improved hepatic steatosis, NAS (>2 points), lobular inflammation, and ballooning. However, the OCA treatment worsened hepatic insulin resistance and increased LDL cholesterol levels. Hence, in another phase 2 clinical trial (Combination Obeticholic Acid (OCA) and Statins for Monitoring of Lipids (CONTROL), NCT02633956, phase 2), a combined treatment of OCA and atorvastatin was carried out and the results showed that atorvastatin could attenuate the OCA-induced increase in LDL cholesterol levels. A phase 3 clinical trial (Study Evaluating the Efficacy and Safety of Obeticholic Acid in Subjects With Compensated Cirrhosis Due to Nonalcoholic Steatohepatitis (REVERSE), NCT03439254, phase 3) to assess the effects of OCA in patients with NASH and fibrosis has recently been completed, while another phase 3 study (Randomized Global Phase 3 Study to Evaluate the Impact on NASH With Fibrosis of Obeticholic Acid Treatment (REGENERATE), NCT02548351, phase 3) is currently underway. The INT-767 is another semi-synthetic FXR agonist. It was reportedly 300 times more potent than the natural homolog [81]. Eight-week treatment of the INT-767 with Lep*^ob/ob^* NASH mice showed improvement in hepatic steatosis, histopathology, ballooning, and inflammation. Hepatic TG content, ALT, and AST levels were also reduced. Notably, the INT-767 revealed superior effects to the OCA in improving the fibrosis stages (36 vs. 82%) [82]. In another pre-clinical study with a high-fat diet (HFD)-rabbit model, the INT-767 attenuated insulin resistance in the visceral adipose tissue and reduced fatty acid synthesis and steatosis [83]. These results supported the hypothesis that the INT-767 may also be considered a potential drug candidate for the treatment of NAFLD. The EDP-305 is a steroidal FXR agonist lacking the carboxylic group associated with the formation of hepatotoxic metabolites (via conjugation with taurine or glycine). It has been reported to possess anti-steatotic, anti-inflammatory, anti-fibrotic, and hepatocyte protective activities. In two NASH mice models, dietary-induced NASH and streptozotocin-HFD mice, the EDP-305 decreased lipogenic gene (SBREP1c and SCD1) levels and hepatic lipid profiles with significant improvement in the NAS [84]. In a phase 2 trial (A Study to Assess the Safety, Tolerability, Pharmacokinetics and Efficacy of EDP-305 in Subjects with Non-Alcoholic Steatohepatitis, NCT03421431, phase 2), 12-week treatment with the EDP-305 led to a 7% reduction in liver fat, but pruritus was reported in about half of the participants.

There have also been developed non-steroidal FXR agonists. For example, cilofexor (GS-9674) is an FXR agonist that does not go through enterohepatic circulation. A pre-clinical study has shown that cilofexor could reduce hepatic fibrosis (with a 37% reduction of Col1α1 expression) and portal hypertension in a NASH rat model [85]. The effects of combination therapy of cilofexor and selonsertib (apoptosis signal-regulating kinase-1 inhibitor) or firsocostat (an acetyl-CoA carboxylase inhibitor) have been clinically investigated in NASH patients with F3-F4 stage compensated cirrhosis (Study to Evaluate the Safety and Efficacy of Selonsertib, Firsocostat, Cilofexor, and Combinations in Participants with Bridging Fibrosis or Compensated Cirrhosis Due to Nonalcoholic Steatohepatitis (NASH) (ATLAS), NCT03449446, phase 2). While cilofexor alone could not meet the trial’s primary outcomes, a significant decrease in NAS (≥2 points), steatosis, lobular inflammation, and ballooning was reported by the cilofexor/firsocostat combination therapy. In addition, improvement in ALT, AST, bile acid, insulin levels, and liver stiffness was also observed. Currently, another phase 2 clinical trial is underway to evaluate the effects of cilofexor/firsocostat and semaglutide combined therapy on NASH patients with F4 compensated cirrhosis (Study of Semaglutide, and the Fixed-Dose Combination of Cilofexor and Firsocostat, Alone and in Combination, in Adults with Cirrhosis Due to Nonalcoholic Steatohepatitis (NASH), NCT04971785, phase 2). Tropifexor (LJN452) is also a non-bile acid FXR agonist. A phase 2 clinical trial has been conducted to evaluate the effects on NASH (Study of Safety and Efficacy of Tropifexor (LJN452) in Patients with Non-alcoholic Steatohepatitis (NASH) (FLIGHT-FXR), NCT02855164, phase 2). Unfortunately, it also did not meet the histological endpoints, and an uncontrolled increase in serum LDL levels was observed as a main side effect. As a different approach, a phase 2 clinical trial with the combination of tropifexor and an SGLT1/2 dual inhibitor, licogliflozin, is currently ongoing to assess the effects on NASH and fibrosis (Efficacy, Safety and Tolerability of the Combination of Tropifexor & Licogliflozin and Each Monotherapy, Compared with Placebo in Adult Patients With NASH and Liver Fibrosis. (ELIVATE), NCT04065841, phase 2). Similarly, a combination of MET409 and empagliflozin is also in a phase 2 clinical trial (Study to Evaluate MET409 Alone or in Combination with Empagliflozin in Patients With Type 2 Diabetes and NASH, NCT04702490, phase 2). Nidufexor (LMB763) is an FXR partial agonist. It serves as a potent modulator of the FXR-dependent genes which eventually leads to a significant reduction in hepatic steatosis, inflammation, and fibrosis with an improvement of the NAS in the STAM^TM^ NASH mice [86]. It was also evaluated in phase 2 clinical trial (Safety, Tolerability, Pharmacokinetics and Efficacy of LMB763 in Patients With NASH, NCT02913105, phase 2). The active clinical trials of FXR agonists for NAFLD are summarized in Table 5.

## 3. Strategies to Enhance the Efficacy of NAFLD Drug Candidates

**Figure 2:**
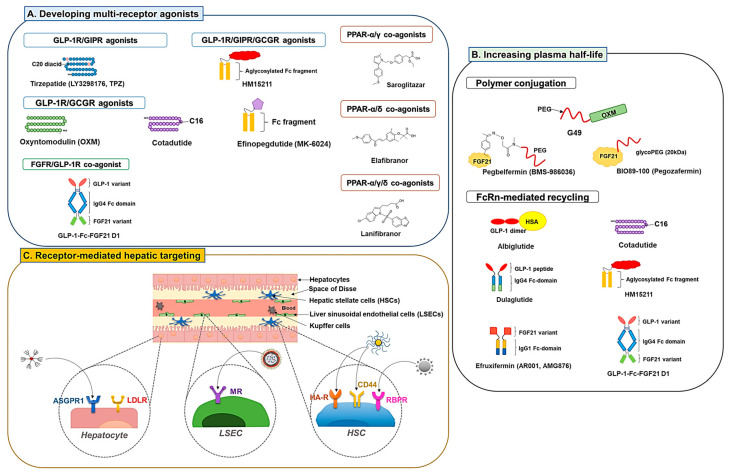
Schematic diagram of the pharmaceutical strategies to make “better drugs” for NAFLD therapy. To enhance the therapeutic efficacy of NALFD drug candidates, the three strategies widely explored include (**A**) the development of multi-receptor agonists, (**B**) engineering long-acting derivatives, and (**C**) devising ligand-decorated nanoparticle-based hepatic targeting systems. The increase of plasma half-life could be accomplished by conjugation of polymers (e.g., PEG) or fusion of IgG Fc or human serum albumin (HSA). The latter enables the drug compound to go through FcRn-mediated recycling. Liver targeting could be achieved by utilizing ligands selective for receptors expressed on the membranes of liver-composing cells (ASGPR, asialoglycoprotein; FcRn, neonatal Fc receptor; FGFR, fibroblast growth factor receptor; GIPR, glucose-dependent insulinotropic polypeptide receptor; GCGR, glucagon receptor; GLP-1R, glucagon-like peptide-1 receptor; HA-R: hyaluronan receptor; HSA, human serum albumin; HSCs, hepatic stellate cells; LDLR, low-density lipoprotein receptor; LSECs, liver sinusoidal endothelial cells; MR, mannose receptor; PEG, polyethylene glycol; PPAR, peroxisome proliferator-activator receptor; RBPR, retinol-binding protein receptor).

As a result of the complexity of NAFLD, the journey to discover an effective drug is far from reaching a conclusion. However, alongside the search for a new entity drug, there has also been growing interest and continuous effort to develop highly efficient strategies to improve the druggability of the discovered drug candidates to achieve the goal. Some of the representative strategies may include (1) developing multiple receptor agonists, (2) engineering long-acting derivatives, and (3) devising ligand-mediated hepatic drug targeting systems. As a result of various issues (including complex pathogenesis, heterogeneity of the disease, insufficient efficacy, or unacceptable toxicity of the drug candidates, etc.), to date, none of the drug candidates have acquired clinical approval. In this regard, the multiple receptor agonists may provide an effective way to synergistically (or additively) enhance the effects of single-agent therapies. Also, some of the extensively studied drug candidates are composed of peptides or proteins, which innately have very short plasma half-lives (except for antibodies and albumin) to sufficiently exert their therapeutic activities. Engineering long-activating derivatives could address this issue and potentially enhance the therapeutic effects of the treatment. Lastly, endowing hepatic targeting ability to the drug candidates could provide them with a larger therapeutic window, allowing greater therapeutic effects with less potential toxicity. A schematic illustration of the strategies is depicted in Figure 2.

### 3.1. Developing Multiple Receptor Agonists

Developing multi-receptor agonists has been a widely adopted strategy for enhanced therapeutic effects in treating metabolic disorders (e.g., obesity, T2DM, and NAFLD). This strategy is believed to provide synergistic or at least additive therapeutic effects by activating multiple receptor signaling. To date, various co-agonists have been engineered that could simultaneously target critical receptors involved in the NAFLD, such as GLP-1R, glucose-dependent insulinotropic polypeptide (GIP) receptor (GIPR), glucagon receptor (GCGR) [87], PPARα, PPARβ/δ, and PPARγ [88,89].

#### 3.1.1. GLP-1R Co-Agonists

GIP is a 42 amino acids long peptide (YAEGTFISDYSIAMDKIHQQDFVNWLLAQKG KKNDWKHNITQ) synthesized by the K cells present in the duodenum and the jejunum [90]. Like GLP-1, GIP serves as an incretin and induces insulin secretion from the pancreatic β cells in response to high glucose levels in the duodenum [91]. GIP also protects the β cells from apoptosis and promotes their proliferation [91]. Furthermore, GIP can regulate the appetite and reduce food intake through its action in the hypothalamus [92]. However, unlike GLP-1, GIP stimulates glucagon secretion and possesses a lipid storage action in the adipose tissue [93]. The GIPRs are present in the CNS and many peripheral organs and tissues (e.g., pancreas, gut, heart, and adipose tissues). A positive correlation between the GIPR level and resistance to obesity appears to exist. Also, T2DM patients often have reduced postprandial levels of GIP secretion [94].

According to the report by Finan, the GLP-1R/GIPR dual-agonists could provide more significant hypoglycemic, hyperlipidemic, and anti-obesity effects compared with single agonists with attenuated gastrointestinal discomfort and nausea (typically associated with GLP-1R agonists) [48]. The most successful case of a dual GLP-1R and GIPR agonist would be the tirzepatide (LY3298176, TPZ) which is a C18 fatty diacid moiety-conjugated peptide with a length of 39 amino acids (YXEGTFTSDYSIXLDKIAQKAFVQWLIAGGPSSGAPPPS; X is α-aminoisobutyric acid (Aib), K20 is acylated with a γGlu-2×OEG linker and the C18 fatty diacid moiety) [95,96]. TPZ has been extensively investigated in 45 clinical trials and was recently approved by the FDA for the treatment of T2DM (Mounjaro^TM^, Eli Lilly and company). In clinical studies, TPZ reduced bodyweight and food intake more significantly than the dulaglutide [93]. Other than T2DM, two clinical trials are ongoing to investigate the effect of NASH treatment (Effect on Non-Alcoholic Fatty Liver Disease in Patients with Type 2 Diabetes Mellitus With Gastric Inhibitory Polypeptide/Glucagon Like Peptide-1 Analogue, NCT05751720, phase 1/2; A Study of Tirzepatide (LY3298176) in Participants With Nonalcoholic Steatohepatitis (NASH) (SYNERGY-NASH), NCT04166773, phase 2).

Glucagon is a 29 amino acids long peptide hormone (HSQGTFTSDYSKYLDSRRAQDFVQWLMNT) secreted from the pancreatic α cells [97,98]. Its production is regulated by various factors (stimulated by hypoglycemia) or substances (inhibited by amylin and insulin) [99]. The primary role of glucagon is to elevate the blood glucose level by promoting gluconeogenesis and glycogenolysis [100]. In addition, glucagon reduces fatty acid synthesis, promotes lipolysis, and regulates cholesterol biosynthesis [101]. Due to these activities, compared with GLP-1R mono-agonists, the GLP-1R/GCGR dual agonists could offer enhanced lipolysis and energy expenditure (via GCGR), as well as improved glucose homeostasis and insulin sensitization (mediated via GLP-1R). Several dual agonists of GLP-1R/GCGR are currently under investigation for their use in the management of NAFLD.

Oxyntomodulin (OXM), a 37 amino acids long peptide hormone (HSQGTFTSDYSKYLDSRRAQDF VQWLMNTKRNRNNIA) secreted by the intestinal L-cells, is an endogenous dual agonist for the GLP-1R and the GCGR. OXM is known to play a crucial role in bodyweight control and glycemic regulation [102], and the treatment of OXM has shown potential effects on bodyweight loss. However, the bioactivity of OXM in activating the GLP-1R and GCGR is lower than both the endogenous GLP-1 and GCG (18- and 50-fold lower in EC50 levels, respectively) [103]. In addition, because of the difference in the activity toward GLP-1R and GCGR, although known as a dual agonist, the metabolic effects of OXM are mainly attributed to GLP-1R agonism [103]. As dual-agonism may provide superior glucoregulatory and anti-obesity effects to mono-agonists [104], there have been attempts to modify the peptide sequence of OXM for more balanced dual agonism for GLP-1R and GCGR. A study by Ma et al. suggests ways to modify OXM [103]. Based on the native OXM sequence, they introduced oppositely charged α-helical favoring amino acid residues (Glu (E) and Lys (K)) in the mid-region of the peptide sequence (S16E, R17K, Q20K, and D21E) to allow them to form salt bridges among themselves to stabilize the conformation. This modification improved activity by 18.9- and 6.4-fold for GLP-1R and GCGR, respectively.

Furthermore, they substituted the C-terminal 8 amino acid residues of the intervening peptide-1 to a truncated exendin-4 sequence, which led to an extra increase in GLP-1R and GCGR activity (1.7- and 1.8-fold, respectively). Overall, with these modifications, the activity of OXM could improve to about 64% activity of both GLP-1 and GCG. Apart from these substitution works for improving the receptor affinity, they also introduced an unnatural amino acid residue (Aib) replacing the DPP-4-hydrolysis liable Ser residue at position 2, and did a Q24C substitution for PEG conjugation. These modifications did not significantly affect OXM activity. However, conjugation of a 40 kDa size PEG to this modified OXM led to a large decrease in the activity (6- and 4-fold reduction of GLP-1R and GCGR activity compared with the non-PEGylated modified OXM).

Other examples of dual GLP-1R/GCGR agonists include cotadutide (MEDI0382) and efinopegdutide (MK-6024, HM12525A). Cotadutide is a palmitoylated glutamate-extended peptide (1′-[palmtoyl-Glu]; HSQGTFTSDKSEYLDSERARDFVAWLEAGG [amide bridge: Glu1′-Lys10]) modified from the native glucagon with balanced agonism toward GLP-1R and GCGR (but still have a 5-fold bias toward GLP-1R activation over GCGR) [105]. The activity for GLP-1R and GCGR was 14.7- and 1.8-fold higher than the OXM. Henderson et al. reported that, in DIO mice, cotadutide produced greater weight loss and comparable hypoglycemic effects to liraglutide. The authors hypothesized that this might be due to the dual action of the drug via GCGR-mediated increased energy expenditure and GLP-1R-mediated reduced food intake. The significant weight loss effect was also found in cynomolgus monkeys [105]. According to Boland et al., cotadutide improved hepatic lipid profiles, as well as glucose tolerance and insulin sensitivity. Moreover, in obese trans-fat-containing amylin liver (AMLN) NASH mice, it could improve hepatic fibrosis and inflammation [106]. In a 52-week phase 2 clinical trial (A Study to Evaluate the Efficacy and Safety of MEDI0382 in the Treatment of Overweight and Obese Subjects with Type 2 Diabetes, NCT03235050, phase 2), significant improvement in lipid profiles, HbA1c, bodyweight, and NAS was observed in cotadutide-treated patients with T2DM and obesity. In the case of efinopegdutide, detailed information on the structure is not informed, but it was reported to possess a balanced activity toward the GLP-1R and GCGR [87]. A phase 2 clinical trial (A Study of Efinopegdutide (MK-6024) in Participants with Nonalcoholic Fatty Liver Disease (NAFLD) (MK-6024-001), NCT04944992, phase 2) was recently completed, and there is another active clinical trial ongoing (A Study to Evaluate the Safety and Efficacy of Cotadutide Given by Subcutaneous Injection in Adult Participants with Non-cirrhotic Non-alcoholic Steatohepatitis with Fibrosis. (PROXYMO-ADV), NCT05364931, phase 2b/3).

HM15211 is a triple agonist for the GLP-1R/GIPR/GCGR. The FDA has granted it a fast-track designation for treating NASH and fibrosis based on promising pre-clinical study results. The efficacy of HM15211 was evaluated in various mice models (DIO, AMLN, or MCD-diet mice). Common among these studies, significant bodyweight loss and improvement in hepatic steatosis were observed [107]. In the NASH mice models, HM15211 revealed superior anti-steatosis and anti-fibrotic effects compared to single agonists (liraglutide, OCA, and selonsertib) [107]. HM15211 was also more effective in reducing bodyweight and hepatic lipid content than liraglutide [108]. In a fructose-fed hamster model, HM15211 showed significant effects on obesity and dyslipidemia. Cell studies suggested that higher clearance of LDL may be the main cause for this improvement, as a significant increase (4.1-fold) of LDLR was observed from the HM15211-treated HepG2 cells [109]. Notably, HM15211 also prevented HSC activation and fibrosis by reducing the production of transforming growth factor- β (TGF-β) and collagen in the HSCs [110]. A phase 1 clinical trial has been completed (A Study of Multiple Doses of HM15211 in Obese Subjects With NAFLD, NCT03744182, phase 1), and a phase 2 clinical trial (Study to Evaluate Efficacy, Safety and Tolerability of HM15211 in Subjects, NCT04505436, phase 2) is currently underway.

Other than the peptidyl agonists, various small molecule-based GLP-1R agonists have recently been discovered. Many are designed to be bispecific for GLP-1R and GIPR or GCGR. Currently, CT-388 is in phase 1 clinical trial for indications of T2DM and obesity (A Study of CT-388 in Otherwise Healthy Overweight and Obese Adults and Patients with Type 2 Diabetes Mellitus, NCT04838405, phase 1), while CT-868 is under a phase 2 trial for T2DM and obesity (A Study of CT-868 in Overweight and Obese Participants with Type 2 Diabetes Mellitus, NCT05110846, phase 2). DD01, a long-acting dual agonist for GLP-1R/GCGR, is also in a phase 1 clinical trial to investigate the effects on NASH, T2DM, and obesity (A Phase 1 Study of DD01 in Overweight/Obese Subjects with T2DM and NAFLD, NCT04812262, phase 1). The GLP-1R dual agonists in active clinical trials for NAFLD are summarized in Table 6.

#### 3.1.2. PPAR Dual and Pan Agonists

PPARs are transcription factors that regulate critical genes involved in the homeostasis of glucose and lipids. Despite different tissue distribution and physiological effects, the three isoforms (PPAR-α, PPAR-β/δ, and PPAR-γ) commonly play critical roles in the regulation of lipid and glucose metabolism [111], which could benefit the treatment of NAFLD [112]. Many PPAR dual and pan agonists have recently been developed and investigated with the expectation of synergistic (or additive) effects for their potential use in NAFLD therapy. The PPAR multi-receptor agonists in clinical trials for NAFLD are summarized in Table 7.

Glitazar is a class of dual agonists with varying degrees of agonism for PPAR-α/γ. Through pre-clinical and clinical studies, their combined effects on dyslipidemia (by PPAR-α) and insulin resistance (by PPAR- γ) have proven their utility in treating metabolic disorders [113]. A representative drug in this class is saroglitazar. This is a marketed drug for treating T2DM and dyslipidemia in India (trade name: Lipaglyn) [114]. It is a PPAR-α/γ dual agonist but with predominant PPAR-α activity. As expected, in clinical studies, saroglitazar could reduce TG, VLDL, and LDL cholesterols, increase HDL cholesterol and improve blood glucose profiles [115]. Regarding NAFLD, pre-clinical studies by Kumar et al. revealed that saroglitazar could provide superior therapeutic effects to pioglitazone on improving not only hepatic steatosis and inflammation, but also fibrosis in the high-fat western diet and lib sugar water (WDSW)-induced NAFLD mice [116]. Based on the successes of pre-clinical studies, there have been eight clinical studies (keyword: “NAFLD” and “saroglitazar”) conducted or currently ongoing (ClinicalTrials.gov identifier: NCT03639623, NCT03617263, 04193982, NCT03061721, NCT02265276, NCT05211284, NCT03863574, NCT05011305). In a phase 2 clinical trial, 16-week treatment of saroglitazar improved the markers of fibrosis and hepatocellular injury and reduced hepatic fat content (Saroglitazar Magnesium in Patients With Nonalcoholic Fatty Liver Disease and/or Nonalcoholic Steatohepatitis (EVIDENCES IV), NCT03061721, phase 2) [89]. Unfortunately, other than saroglitazar, most glitazars have failed during development because of relevant adverse events, including potential carcinogenic or cardiovascular, renal, or bone marrow toxicity issues [113].

Elafibranor (GFT505) is a PPAR-α/δ dual agonist with preferential activity on the PPAR-α. It was investigated early for potential use in T2DM and hyperlipidemia, but has recently drawn more interest in treating NAFLD. According to the pre-clinical studies by Staels et al., elafibranor induced a significant reduction in hepatic gene expression related to inflammation (IL-1β, TNF-α, and F4/80) and fibrogenesis (TGF-β and TIMP-2), leading to reduction of steatosis, inflammation, and fibrosis [117]. In a phase 2b clinical trial (Phase IIb Study to Evaluate the Efficacy and Safety of GFT505 Versus Placebo in Patients With Non-Alcoholic Steatohepatitis (NASH), NCT01694849, phase 2), the elafibranor could resolve NASH in NAS over four patients with no worsening of hepatic fibrosis [118]. Unfortunately, a phase 3 trial for NASH was recently terminated due to limited efficacy (Phase 3 Study to Evaluate the Efficacy and Safety of Elafibranor Versus Placebo in Patients with Nonalcoholic Steatohepatitis (NASH) (RESOLVE-IT), NCT02704403, phase 3).

Lanifibranor (IVA337) is a PPAR-α/γ/δ pan agonist. According to a pre-clinical study by Lefere et al., lanifibranor could provide a superior anti-NAFLD activity than single agonists (fenofibrate (PPAR-α), pioglitazone (PPAR-γ) and GW501516 (PPAR-δ)) in different NAFLD mice models [119]. Notably, synergistically combined effects of the single agonists were realized in lanifibranor-treated mice. The study results suggested improvement of hepatic steatosis by PPAR-α activation, reduction of hepatic inflammation and macrophage activation by PPAR-δ, and deactivation of HSCs by PPAR-γ [119]. In a completed phase 2 clinical trial with active NASH patients (Phase 2b Study in NASH to Assess IVA337 (NATIVE), NCT03008070, phase 2), lanifibranor treatment significantly improved the steatosis activity fibrosis (SAF) score and decreased liver enzyme levels and biomarkers of lipid, inflammation, and fibrosis [88]. Three clinical trials (including 2 phase 3) are currently ongoing with lanifibranor to evaluate the therapeutic efficacy in NAFLD (Placebo-controlled, Proof-of-concept Study to Evaluate the Safety and Efficacy of Lanifibranor Alone and in Combination With SGLT2 Inhibitor EmpaGliflozin in patiEnts with NASH and Type 2 Diabetes Mellitus (LEGEND), NCT05232071, phase 2; Lanifibranor in Patients With Type 2 Diabetes & Nonalcoholic Fatty Liver Disease, NCT03459079, phase 2 and A Phase 3 Study Evaluating Efficacy and Safety of Lanifibranor Followed by an Active Treatment Extension in Adult Patients With (NASH) and Fibrosis Stages F2 and F3 (NATiV3), NCT04849728, phase 3). Active clinical trials of PPAR multi-receptor agonists are summarized in Table 7.

#### 3.1.3. FGFR Dual Agonists

Interest in FGFR/GLP-1R dual agonists as potential NAFLD therapeutics has been growing recently. According to Liu et al., there appears to be a synergistic interplay loop among the FGFR and GLP-1R agonists. It was found that FGF21 mediates GLP-1R’s effects on inhibiting the hepatic glucose output, while the hepatic production of FGF21 could be upregulated by GLP-1R agonists (e.g., exenatide and liraglutide) [120]. YH25724 and GLP-1-Fc-FGF21 D1 are long-acting dual agonists against FGFR and GLP-1R. Both have shown promising effects in pre-clinical studies. In MCD-diet mice, YH25724 more significantly alleviated hepatic fibrosis and inflammation than single agonists (Fc-GLP-1, Fc-FGF21, and dulaglutide). Consistently, in DIO mice, a significantly higher reduction in hepatic steatosis, plasma TG levels, and bodyweight was achieved by YH25724, compared with the single agonists [121]. GLP-1-Fc-FGF21 D1, developed by Pan et al. is a long-acting GLP-1R/FGFR dual agonist that consists of an FGF21 with improved β-klotho binding property [122]. The HFD-fed ob/ob mice treated with GLP-1-Fc-FGF21 D1 provided a significantly higher hypoglycemic effect with a more significant bodyweight reduction than single agonists. Furthermore, GLP-1-Fc-FGF21 D1 also offered more significant therapeutic effects in attenuating NASH progression regarding hepatic function, lipid profiles, and NAS scores than the single agonists.

### 3.2. Development of Long-Acting Derivatives

A bottleneck challenge for the clinical application of active proteins (or peptides) is presented by their plasma half-lives, which are too short. Different strategies have been adopted to improve their pharmacokinetic (PK) profiles, including polymer conjugation and fusion with albumin or the immunoglobulin G (IgG) Fc domain.

#### 3.2.1. Polymer Conjugation

The polymer conjugation strategy mainly relies on increasing the protein’s hydrodynamic radius to retard their glomerular filtration, as well as physically interfere with the uptake by phagocytic or endothelial cells. Various polymers have been explored for modifying protein therapeutics. The most widely adopted polymer is polyethylene glycol (PEG), and there are also intrinsically disordered inert large polypeptide repeats, such as XTEN, PAS (proline, alanine, Serine), and ELP (elastin-like polypeptide) [123].

Despite possessing potential anti-obesity activity, the short plasma half-life (12 min) of OXM remains a challenge to fully realize its effects [124]. Ma et al. also reported the study of a PEGylated OXM analog with amino acid substituted for increased GCGR and GLP-1R activity (by 2- and 8-fold, respectively). The 40 kDa size PEG conjugation extended the plasma half-life of the OXM analog to 19.9–25.2 h (in mice). Seven weeks of once-weekly treatment of the modified OXM analog in *db/db* mice could provide more significant improvement in insulin sensitivity and glucose tolerance with reduced plasma TG and cholesterol levels than liraglutide. Furthermore, in diet-induced obese (DIO) mice, a more significant reduction in body fat mass and hepatic lipid content was also achieved compared to liraglutide [103].

There have also been many efforts to develop PEGylated FGF21, as the short plasma half-life of FGF21 (0.7–1.1 h in mice) limits its therapeutic use [58]. A PEGylated variant of FGF21, B1344, was developed by Ye et al. [125]. To increase the potency of human FGF-21, they carried out an interesting approach, replacing a functional domain of the human FGF-21 with that of the mouse FGF21 and insertion of an alanine residue at the N-terminal site [125]. The FGF-21 mutant (ahmFGF-21) was further PEGylated at the N-terminal site with a 20 kDa mPEG-propionaldehyde. The PEGylated-ahmFGF-21 revealed an extended plasma half-life, reduced immunogenicity, and improved hypoglycemic and hyperlipidemic effects [125]. When the B1344 was tested in MCD-diet-induced NASH mice and obese cynomolgus monkeys, significant effects were observed regarding reduced hepatic fat content, bodyweight, plasma lipid profiles, hepatic steatosis, inflammation, and fibrosis [126].

Notably, there are reports of different approaches to achieve site-specific PEGylation of the FGF21. Xu et al. reported a systematic study to engineer site-specific mono- and dual-PEGylated FGF21 that retain good activity but cause minimal vacuole formation (a potential risk caused by PEG). To achieve this goal, they produced FGF21 mutants by introducing cysteine residues at selected positions in the protein sequence (based on an FGFR1c binding model) and chemically conjugated them with sulfhydryl-reactive PEG. The results revealed that the PEGylation site could significantly affect the activity of FGF21, and the PEG configuration and number could also influence the vacuologenesis [127].

Pegbelfermin (BMS-986036) is another example of a long-acting FGF21, synthesized by site-specific conjugation of a linear 30 KDa size PEG to the FGF21 mutant introduced with an unnatural *p*-acetyl phenylalanine (*p*AcF) residue. The PEGylation could significantly extend the plasma half-life of FGF21 to 14.7–33.9 h in rats. In a pre-clinical study, twice-weekly treatment of the PEGylated FGF21 improved lipid profiles, pancreatic function, and insulin sensitivity [128]. Pegbelfermin has also been investigated in a series of clinical studies [129]. In a 16-week phase 2a clinical trial, once-a-week treatment with pegbelfermin significantly reduced hepatic fat content in NASH patients (A Study of Experimental Medication BMS-986036 in Adults With Nonalcoholic Steatohepatitis (NASH) and Liver Cirrhosis (FALCON 2), NCT03486912, phase 2) [130]. In a different phase 2 clinical trial, pegbelfermin significantly improved the high-density lipoprotein (HDL) cholesterol, markers of fibrogenesis neoepitope-specific N-terminal pro-peptide of type III collagen (PRO-C3) in patients with diabetes and obesity (A Study to Evaluate BMS-986036 in Obese Adults With Type-2 Diabetes, NCT02097277, phase 2) [131]. Recently, two phase 2b clinical trials (A Study of Experimental Medication BMS-986036 in Adults With Nonalcoholic Steatohepatitis (NASH) and Liver Cirrhosis (FALCON 2), NCT03486912, phase 2 and A Study of Experimental Medication BMS-986036 in Adults With Nonalcoholic Steatohepatitis (NASH) and Stage 3 Liver Fibrosis (FALCON 1), NCT03486899, phase 2) have been carried out to evaluate the therapeutic effects of pegbelfermin in NASH and liver cirrhosis [132].

Pegozafermin (BIO89–100) is a site-specific glycoPEGylated recombinant FGF21 analog with an extended half-life of 55–100 h (in humans), potentially allowing once weekly or fortnightly dosing [133,134]. Pegozafermin is prepared by chemically conjugating a 20 kDa size linear PEG to an FGF21 mutant, mediated by a glycosyl linker. In a phase 1b/2a trial (A Multiple Ascending Dose Study of BIO89–100 in Subjects With Biopsy Confirmed NASH or NAFLD and at High Risk of NASH, NCT04048135, phase 1b/2a), 12-week treatment with the pegozafermin significantly reduced hepatic volume and fat fraction and improved NAFLD-related markers (TG, LDL-C, HDL-C, non-HDL-C, adiponectin, bodyweight, ALT, and PRO-C3) [135]. A 24-week phase 2 clinical trial (Study Evaluating the Safety, Efficacy and Tolerability of BIO89–100 in Subjects with Biopsy-confirmed Nonalcoholic Steatohepatitis (NASH) (ENLIVEN), NCT04929483, phase 2) is ongoing, with NASH patients in F2/F3 fibrosis stage.

Apart from PEGylation, conjugation of ELPs has also been employed for extending the plasma half-life of FGF21. The ELPs are peptide polymers composed of a repetitive (VPGX_aa_G) sequence (X_aa_: any amino acid residues besides proline) [136]. Notably, they are thermally responsive and possess a tunable transition temperature that could be modulated by their X_aa_ residue and molecular size. By elaborately designing the ELP-drug sequence to have an adequate transition temperature (27–32 °C), Gilroy et al. could develop a GLP-1-ELP-FGF21 fusion protein that forms a depot after subcutaneous injection and released in a sustained-release fashion (absorption half-life: 7.6 days) [137]. The GLP-1-ELP-FGF21 fusion protein design consisted of three components: GLP-1 at the N-terminus, FGF21 at the C-terminus, and ELP in the middle. The presence of ELP between the GLP-1 and FGF21 provided flexibility, ensuring dual receptor activity. The GLP1-ELP-FGF21 showed comparable activity for GLP-1R and FGFR to the ELP-GLP1 and ELP-FGF21 [137]. However, probably due to additive (or synergistic) effects, treating GLP1-ELP-FGF21 to db/db mice provided a more significant glycemic control and bodyweight reduction compared to monotherapy or the treatment of GLP1/FGF21 mixture.

#### 3.2.2. Exploiting Neonatal Fc Receptor (FcRn)-Mediated Recycling

Serum proteins such as albumin and IgG have a relatively long plasma half-life (generally several weeks), which is attributed to FcRn-mediated recycling. FcRn shares structural similarities with the MHC class I molecule, and is expressed in many tissues and organs, such as the brain, heart, kidneys, and gut. FcRn was first recognized as the carrier molecule for the transport of IgG from the mother to the fetus [138], but later, it was found that the FcRn plays a crucial role in extending the plasma half-life of the IgG and albumin [139]. FcRn-mediated recycling relies on the pH-dependent binding of IgG (via the Fc region) and albumin with the FcRn. When the IgG or albumin in the bloodstream internalizes endothelial cells, high-affinity binding with the FcRn occurs at acidic pH (<6.5) in the endosomes. FcRn binding allows protection from enzymatic degradation and lets the IgG or albumin get recycled back to circulation (or transcytose to the other side), while other molecules unbound to FcRn become degraded in the lysosomes [138]. This FcRn-mediated recycling has been successfully exploited in many protein therapeutics by directly conjugating them with albumin or IgG (specifically the Fc region). An alternative method is to couple the proteins with albumin-binding moieties (e.g., lipids), which allows the formation of non-covalent complexes with circulating albumins after administration.

Human serum albumin (HSA), produced by the liver, is the most abundant plasma protein (accounting for around 50% of proteins in the plasma [140]. It comprises three homologous domains, with each domain made up of two subdomains. HSA exhibits a strong pH-dependent binding affinity toward the FcRn, enabling it to utilize the FcRn-mediated recycling effectively [141]. FcRn binding occurs at the hydrophobic cavities located between subdomains IIA and IIIA of HSA. The high plasma concentration and an extended plasma half-life render albumin to become an effective carrier. Furthermore, HSA can bind to various plasma substances, including fatty acids. The crystallographic investigation confirmed albumin binding with different size fatty acids and the presence of multiple binding sites [142]. These findings led to the development of long-acting lipid-conjugated protein therapeutics.

For the GLP-1R agonists, different approaches have been attempted to utilize the FcRn-mediated recycling, and some of the long-acting modified agonists have successfully reached clinical approval. A straightforward strategy to achieve the goal was directly fusing human serum albumin (HSA) to the drug candidate. For example, albiglutide is an approved long-acting GLP-1 fusion protein consisting of a modified GLP-1(7–36) dimer and human serum albumin. For improved resistance against DDP-4, the albiglutide has A8G substitutions for both GLP-1s. The plasma half-life of albiglutide is 5 days (in human) [143]. A strategy that has been more widely adopted recently is the development of lipid-conjugated drugs. Liraglutide and semaglutide are good examples of lipid-conjugated long-acting GLP-1 analogs with high albumin binding efficacy. Liraglutide has a similar peptide backbone to GLP-1(7–37), but differs by a K34R substitution and covalent conjugation of a palmitic acid (C16) to the acylated K26 position via a γ-glutamic acid spacer [144]. In the case of semaglutide, compared with liraglutide, it has an additional substitution at A8 with an unnatural 2-aminobutyric acid (Aib) for protection from DDP-4-induced degradation. Semaglutide also possesses an octadecanoic (C18) diacid moiety conjugated at K26 via a “γGlu-2xOEG” linker [145]. The reported plasma half-lives of liraglutide and semaglutide are 11–15 h and 1 week, respectively (plasma half-life of GLP-1: 1.5–5 min) [145,146,147]. The GLP-1R/GCGR dual agonist, cotadutide, is also made up of a palmitoylated 31 amino acids long peptide, and the its plasma half-life in T2DM patients was 12.9 h [148].

Conjugation of proteins to the IgG Fc domain is also a widely adopted strategy to exploit FcRn-mediated recycling. The FcRn-binding site of the IgG Fc domain, positioned at the interface of CH2/CH3 domains, is conserved among different species [149]. The notable pH dependency of the IgG/FcRn binding is primarily attributable to the histidine residues on both sides. The histidine residues on the FcRn contribute to greater stability at acidic pH, while those on the IgG (e.g., H310 and H433 for IgG4) are directly engaged in salt bridge formation with the counterparts of FcRn [150]. On the FcRn, the IgG has a distinct binding site that is non-overlapping with albumin [141]. Dulaglutide is a good example of the class of IgG Fc-coupled drugs. Dulaglutide, an FDA-approved T2DM therapeutics, is a recombinant fusion protein consisting of two identical DPP-4-resistant modified GLP-1 (7–37), separately fused to each arm of modified IgG4 Fc domain via peptide linkers. The modified GLP-1 (7–37) composing the dulaglutide has an amino acid substitution at three positions (A8G, G22E, and R36G). Its long plasma half-life (90–95 h) makes it suitable for once-weekly s.c. administration [151]. Efinopegdutide (MK-6024, HM12525A) is a chemical conjugate of a synthetic GLP-1/GCG peptide with a human IgG Fc via a PEG linker [87]. This efinopegdutide also has a long plasma half-life of 112.5–276.2 h (with s.c. administration in humans). Similarly, HM15211 is an IgG Fc domain conjugate of GLP-1R/GIPR/GCGR triple co-agonist linked by a flexible PEG linker. Its plasma half-life is 72.09–142.10 h (with s.c. administration in humans) [152].

Efruxifermin (AR001, AMG876, Fc-FGF21[RGE]) is a fusion protein composed of a human IgG1 Fc domain and two modified FGF21s that have amino acid substitutions at three positions (L98R, P171G, and A180E) for reduced aggregation, protection from proteolytic degradation and increased binding affinity to β-klotho [153]. The reported plasma half-life of efruxifermin is 3–3.5 days (in humans) [154]. Efruxifermin was clinically investigated for NASH therapy [154]. Sixteen weeks of efruxifermin treatment (A Study of Efruxifermin in Subjects with Histologically Confirmed Nonalcoholic Steatohepatitis (NASH), NCT03976401, phase 2) provided a significant reduction in the hepatic fat fraction (HFF), serum ALT levels and improvement in NAS (≥2 scores) without worsening of fibrosis. Reduction in fibrosis and hepatic injury markers by efruxifermin treatment was also reported [155]. Currently, two phase 2 trials (A Study of Efruxifermin in Subjects with Compensated Cirrhosis Due to Nonalcoholic Steatohepatitis (NASH) (Symmetry), NCT05039450, phase 2 and A Study of Efruxifermin in Non-Cirrhotic Subjects With Histologically Confirmed Nonalcoholic Steatohepatitis (NASH) (Harmony), NCT04767529, phase 2) are underway. Pan et al. developed a group of mutant FGF21 fusion proteins [122]. While screening for mutants with enhanced affinity to β-klotho, they also constructed long acting-FGF21 fusion proteins (composed of IgG4 Fc and the FGF21 mutant) and, further, GLP-1-Fc-FGF21 fusion proteins. In vitro assay results confirmed GLP-1-Fc-FGF21 fusion proteins possessing higher binding affinity toward the β-klotho and greater potency for downstream signal activation than a single agonist or a mixture. Animal studies also revealed more potent anti-diabetic and anti-obesity activity by the GLP-1-Fc-FGF21 fusion protein than single agonists. Notably, the plasma half-life of the GLP-1-Fc-FGF21 was 30.3 h (in mice) and 25.9 h (in rats).

### 3.3. Receptor-Mediated Hepatic Targeting

A major challenge of hepatic drug delivery for the treatment of NAFLD is that only a small portion of the administered drug may reach the liver. An effective strategy to overcome this issue may be exploiting a nano-carrier, as the major distribution/elimination site of most of the NPs is the liver [156]. However, even though the NPs could successfully reach the liver, they would generally end up cleared by the Kupffer cells (KCs). Phagocytosed NPs by KCs would be eliminated from the system without eliciting any effects [157]. Other factors (e.g., size, surface charge, pathological status, etc.) could significantly affect the fate of the delivered NPs in the liver [158]. For example, the fenestrae of the LSECs allow the penetration of substances smaller than 200 nm [159]. When Park et al. investigated the hepatic distribution of PLGA NPs (particle size: 271 nm and zeta potential: −28.3 mV), most of the NPs were found in non-parenchymal cells (KCs and LSECs), with 15% of the NPs accumulated in KCs, 20% in LSECs, 1% in HSCs, and only 4% in hepatocytes [160]. For cell-specific targeting, there have been continuous research efforts to develop drug-loaded NPs decorated with ligands that could selectively bind to receptors expressed on different liver cell types. Such receptors that have been widely adopted for recent studies include the asialoglycoprotein receptor (ASGPR), hyaluronan receptor (HA-R), low-density lipoprotein receptor (LDLR), mannose receptor (MR), and retinol-binding protein receptors (RBPR).

ASGPR is a C-lectin-type scavenger receptor mainly present in hepatocytes [161]. The ASGPR clears up desialylated serum glycoproteins, and ligands with exposed terminal N-acetylgalactosamine (GalNAc) or galactose (Gal) residues could also be taken up by the ASGPR [161]. Owing to the selectivity toward galactose derivatives, high expression in hepatocytes, and induction of robust internalization, ASGPR-mediated targeting has been considered an appealing strategy for hepatic drug delivery [162]. Indeed, Bon et al. reported a significantly higher hepatic distribution of anti-ASGPR antibody (42% I.D./g tissue at 8 h post-administration) over the control antibody (4.8% I.D./g tissue) [163]. Consistently, Sharma et al. reported that a novel galactose dendrimer (GAL-24) could be preferentially localized to the liver [164]. Upon administration of GAL-24 to the mice, significant liver accumulation of 20% ID in 1 h and 80% ID in 24 h was reported. Notably, about 80% of the hepatocytes were positive for GAL-24, with minimal off-target accumulation and systemic toxicity.

Hyaluronan (HA) is a polymer that is an essential component of the extracellular matrix. The HA is mainly cleared by the lymph node but could also be cleared through HA-R-mediated hepatic clearance [165]. The HA-R has two isoforms, stablin-1 (Stab1) and stabilin-2 (Stab2). Among them, Stab2 is the primary receptor involved in HA clearance. Stab2 is highly expressed in the liver, lymph nodes, and spleen. Apart from HA-R, HA could also bind CD44 and mediate targeting to CD44 over-expressed activated HSCs. Exploiting HA-R/CD44 for liver targeting, Yang et al. developed a conjugate of tumor necrosis factor-related apoptosis-inducing ligand (TRAIL) and HA (HA-TRAIL) to treat liver fibrosis. When administered to an N-nitrosodimethylamine-induced hepatic fibrosis SD rat model, HA-TRAIL showed a significantly higher delivery and a prolonged residency in the liver, compared to the unmodified TRAIL (>4 days) after single i.v. administration [166]. Li et al. developed a deoxycholic acid-modified HA-conjugated micelle loaded with silibinin [167]. Compared with the nonspecific micelle, the HA-conjugated micelle showed an HSC-specific delivery in the fibrotic liver and led to a more significant improvement of hepatic fibrosis condition in a CCl_4_-induced fibrosis rat model.

Retinol binding protein 4 (RBP4) is a transporter protein belonging to the lipocalin family [168]. This RBP4 is mainly produced from the liver (esp. in HSCs) and circulates through the blood. It serves a significant role in vitamin A (VA) transportation and cellular uptake [169]. Notably, HSCs are a major site for retinol storage. Approximately 80% of the total retinol is taken up from the blood circulation and stored in HSCs via the help of retinol-binding protein receptors (RBPRs) [170]. Considering the presence of RBPRs in HSCs, retinol has often been exploited as a ligand for HSC targeting. For example, Qiao et al. developed a retinol-modified polymeric micelle, poly(lactide-*co*-glycolide)-polyspermine-poly(ethylene glycol)-retinol (PLGA-PSPE-PEG-VA) loaded with silibinin and Col1α1 siRNA (siCol1α1) (“CGPVM”). CGPVM highly accumulated in the liver (especially for the fibrotic liver) and showed the greatest anti-fibrotic effects among the experimental groups [171]. Hassan et al. developed a retinol-modified chitosan NP loaded with JQ1 and atorvastatin. They prepared low (0.59 retinol/nm^2^) and high (0.86 retinol/nm^2^) density retinol-tagged NPs and examined their HSC uptake and liver accumulation profiles in healthy and CCl_4_-induced hepatic fibrosis mice. Interestingly, compared with the unmodified NP, the low-density retinol-tagged NP showed significantly higher (2.7-fold) uptake in HSC than the HEK 293 cells but not the high-density retinol-tagged NP. Furthermore, only the low-density retinol-tagged NP revealed significantly higher (2-fold) liver accumulation in the fibrotic mice compared with the healthy mice. The authors suggested that the higher density of retinol on the surface of NP might have caused steric hindrance to the interaction between the NPs and RBPs [172].

MR, also known as cluster of differentiation 206 (CD206), is a type I transmembrane receptor belonging to the family of C-type lectin family. The MR is present on the surface of various cells (e.g., macrophages, dendritic cells, and LSECs). Specifically, it was reported that there are present 20,000–25,000 MRs per cell on the LSECs [173,174]. The representative ligands of the MR include mannose, N-acetylglucosamine, and N-acetylgalactosamine (GalNAc) [175]. MR has been considered a favorite target for hepatic drug delivery because of the presence of MR in LSECs. Kim et al. recently reported interesting data regarding cell-specific targeting in the liver [176]. They evaluated the cell uptake and transfection of mRNA-loaded mannose-decorated LNPs. The results suggested that the lipid-PEG content, particle size, and a ligand (mannose) could be critical in targeting efficiency. With 1.5% PEG-lipid but without mannose, the mRNA transfected cells were 80, 40, and 10% for hepatocytes, LSECs, and Kupffer cells, respectively. With an increase in the PEG-lipid content, the difference in transfected cell population became smaller between the hepatocytes and LSECs, while it got larger at lower PEG-lipid content. They said that the higher PEG content may have reduced the apolipoprotein E (ApoE)-mediated cellular uptake of the LNPs by interfering with the adsorption of ApoE. Notably, with the decoration of mannose on the particles, the transfected cell ratios dramatically changed for the hepatocytes and LSECs (hepatocytes: 70 to 15%, and LSECs: 15 to 70%). The results further implied that MR-mediated targeting could be a powerful tool for selective drug delivery to the LSECs [176].

LDLR is a cell surface endocytic receptor that mediates the uptake of low-density lipoproteins (LDL) rich in cholesterol. It recognizes apolipoprotein B100 (ApoB100) and ApoE, which are embedded in LDL particles and chylomicron/very low-density lipoprotein remnants, respectively. LDLR is highly expressed in the liver and regulated by sterol regulatory element binding protein-2 (SREBP-2) [177]. Various approaches have been attempted to exploit the high affinity of LDLR to the ApoB100 and ApoE for hepatic drug delivery. Wang et al. developed an ApoB100-decorated lipid NP loaded with anticancer drugs sorafenib and dihydroartemisinin to target hepatic cancer cells. The ApoB100-decorated NPs showed significantly higher uptake in the liver cancer cells than the control NPs [178]. Through cellular analysis on *ldlr*^−/−^ hepatocytes, Akinc et al. demonstrated that siRNA-loaded lipid NP (LNP) could internalize hepatocytes by the ApoE-LDLR pathway. An ionizable lipid, DLinMCD3MA (MC3), was also reported to have hepatocyte homing properties mediated by the affinity of ApoE to the LDLR [179]. In 2018, the FDA approved an siRNA NP formulation based on a second-generation MC3-LNP for treating polyneuropathies named ONPATTRO (ALN-TTR01).

## 4. Conclusions and Future Perspectives

Extensive research has been conducted to discover effective therapeutics for treating NAFLD. A broad spectrum of drug candidates has been studied, and a group has entered clinical investigations. Various pharmaceutical strategies, including the development of co-agonists or long-acting drugs and devising active hepatic targeting techniques, have been explored to improve the therapeutic efficacy of the drugs. There has not yet been any significant success in clinical trials, and the journey to reach the final goal to discover sufficiently effective drugs for NAFLD is far from complete. However, with the continuous research efforts to elucidate the underlying mechanism of the disease and develop effective pharmaceutical strategies, there are growing expectations for the advent of effective and safe drugs for treating NAFLD in the near future.

## Figures and Tables

**Table 1:** Active clinical trials of SGLT2 inhibitors for NAFLD.

No.	Molecules	Development Stage	Clinical Trial Identification	Estimated Year of Completion
1	Canagliflozin	N/A	NCT05422092	2023
		N/A	NCT05513729	2024
2	Dapagliflozin	Phase 3	NCT03723252	2023
			NCT05308160	2024
		Phase 4	NCT05459701	2023
			NCT05140694	2025
			NCT05254626	2025
3	Empagliflozin	N/A	NCT05694923	2024
		Phase 2	NCT03867487	2024
			NCT05140694	2025
		Phase 3	NCT05605158	2024
		Phase 4	NCT04642261	2023
			NCT04976283	2023
			NCT04639414	2023
			NCT05140694	2025
4	Ertugliflozin	Phase 4	NCT05644717	2024

**Table 2:** Active clinical trials of GLP-1R agonists for NAFLD.

No.	Molecules	Development Stage	Clinical Trial Identification	Estimated Year of Completion
1	Dulaglutide	Phase 4	NCT03648554	2024
2	Liraglutide	N/A	NCT05779644	2025
3	Semaglutide	Phase 2	NCT04216589	2023
			NCT03884075	2024
			NCT04971785	2024
			NCT05016882	2024
		Phase 3	NCT05067621	2027
			NCT04822181	2029
		Phase 4	NCT04639414	2023
			NCT05813249	2024

**Table 3:** Active clinical trials of PPAR agonists for NAFLD.

No.	Molecules	Target	Development Stage	Clinical Trial Identification	Estimated Year of Completion
1	Pioglitazone	PPAR-γ	Phase 2	NCT04501406	2027
			Phase 3	NCT05605158	2024
			Phase 4	NCT04976283	2023
				NCT05305287	2027

**Table 4:** Active clinical trials of fibroblast growth factor (FGF) analogs for NAFLD.

No.	Molecules	Structure	Development Stage	Clinical Trial Identification	Estimated Year of Completion
1	Aldafermin (NGM282)	FGF19 analog	Phase 2	NCT04210245	2023
2	bFKB1 (BFKB8488A)	FGFR1c/β-klotho bispecific antibody	Phase 2	NCT04171765	2023
3	Efruxifermin (AR001)	Fc-FGF21 analog	Phase 2	NCT05039450	2024
				NCT04767529	2024
4	MK-3655 (NGM313)	FGFR1c/β-klotho bispecific antibody	Phase 2	NCT04583423	2023
5	Pegozafermin (BIO89–100)	glycoPEGylated FGF21	Phase 2	NCT04929483	2023

**Table 5:** Active clinical trials of FXR agonists for NAFLD.

No.	Molecules	Development Stage	Clinical Trial Identification	Estimated Year of Completion
1	Cilofexor + semaglutide and firsocostat	Phase 2	NCT04971785	2024
2	OCA, 6-α-ethyl-chenodeoxycholic acid (6-ECDCA, INT-747)	Phase 2	NCT05573204	2024
		Phase 3	NCT02548351	2025
3	MET409	Phase 2	NCT04702490	2022
4	Tropifexor + licogliflozin	Phase 2	NCT04065841	2024

**Table 6:** Active clinical trials of GLP-1R dual/triple agonists for NAFLD.

No.	Molecules	Target	Development Stage	Clinical Trial Identification	Estimated Year of Completion
1	Cotadutide (MEDI0382)	GLP-1R/GCGR	Phase 2/3	NCT05364931	2024
2	DD01	GLP-1R/GCGR	Phase 1	NCT04812262	2022
3	HM15211	GLP-1R/GIPR/GCGR	Phase 2	NCT04505436	2024
4	Tirzepatide (LY3298176, TPZ)	GLP-1R/GIPR	Phase 1/2	NCT05751720	2025
			Phase 2	NCT04166773	2024

**Table 7:** Active clinical trials of PPAR multi-receptor agonists for NAFLD.

No.	Molecules	Target	Development Stage	Clinical Trial Identification	Estimated Year of Completion
1	Lanifibranor	PPAR-α/γ/δ agonist	Phase 2	NCT05232071	2023
				NCT03459079	2024
			Phase 3	NCT04849728	2026
2	Saroglitazar	PPAR-α/γ agonist	Phase 2	NCT03617263	2023
				NCT03639623	2023
				NCT05011305	2023
				NCT05211284	2025

## Data Availability

Not applicable.
